# Sleep Disturbances as a Statistical Mediator Between Perceived Academic Stressors and Emotional, Cognitive, and Physical Stress Responses in University Students

**DOI:** 10.3390/bs16071186

**Published:** 2026-07-14

**Authors:** Cristina Ruiz-Camacho, Margarita Gozalo

**Affiliations:** 1 Department of Psychology and Anthropology, Faculty of Education and Psychology, University of Extremadura, 06071 Badajoz, Spain; cristinarc@unex.es; 2 Department of Psychology and Anthropology, Faculty of Sport Science (Psychology Laboratory), University of Extremadura, 10005 Caceres, Spain

**Keywords:** academic stressors, sleep disturbances, irritability, negative thoughts, physical exhaustion, statistical mediation

## Abstract

Sleep disturbances are central to students’ experience of academic stress, yet their involvement in differentiated emotional, cognitive, and physical responses to academic demands requires further specification. This study tested a statistical mediation model in which sleep disturbances were specified as the statistical mediator between perceived academic stressors and irritability, negative thoughts, and physical exhaustion. Using a non-experimental cross-sectional design, data were collected from 1014 undergraduates at the University of Extremadura (M_age_ = 20.56, SD = 3.50), who completed the Academic Stressors Scale (E-CEA) and Academic Stress Responses Scale (R-CEA). Three mediation models were estimated with PROCESS Model 4, adjusting for gender, year of study, and field of study. Academic stressors were positively associated with sleep disturbances (β = 0.52, *p* < 0.001), which were positively associated with the three stress responses (β = 0.30–0.41, all *p* < 0.001). Academic stressors remained directly associated with these outcomes after accounting for sleep disturbances (β = 0.32–0.48, all *p* < 0.001). Indirect associations through sleep disturbances were observed for irritability (β = 0.19, 95% CI [0.15, 0.23]), negative thoughts (β = 0.16, 95% CI [0.12, 0.20]), and physical exhaustion (β = 0.21, 95% CI [0.17, 0.25]). Findings indicate partial cross-sectional statistical mediation and suggest that sleep-focused prevention may complement efforts to manage academic demands and student well-being.

## 1. Introduction

The university context constitutes a particularly relevant setting for the study of academic stress, as students are required to respond continuously to demands related to assessment, workload, time pressure, performance expectations, and adaptation to the conditions of the teaching–learning process. These demands may become stressors when they are appraised as relevant, persistent, or difficult to manage with the available resources. The previous literature has shown that academic stress is associated with lower psychological well-being, poorer academic adjustment, and a higher risk of emotional symptoms among university students (Barbayannis et al., 2022; Karyotaki et al., 2020; Reddy et al., 2018). Beyond psychological symptoms, academic stress is also relevant to students’ academic trajectories, as difficulties in adaptation and persistence have been linked to dropout-related processes in higher education (Buizza et al., 2024; Simón & Puerta, 2022). Within this framework, academic stressors have been described as central elements for understanding students’ distress, particularly when they are linked to assessment situations, overload, beliefs about performance, or difficulties in organizing academic tasks (Cabanach et al., 2016; Estrada-Araoz et al., 2024).

The relationship between academic stressors and university students’ distress does not follow a uniform pattern. Students may express stress through diverse responses, depending on the nature of the stressor, its intensity and duration, and the personal or contextual resources available. From transactional and systemic-cognitive models of stress, distress emerges when the demands of the academic environment are appraised as demanding or difficult to manage, generating an imbalance in the student’s relationship with that environment, which may be reflected in psychological, physical, and behavioral indicators (Barraza Macías, 2006; Lazarus & Folkman, 1984). In line with this view, previous studies have linked academic stress to different indicators of distress, including exhaustion, sleep disturbances, irritability, and negative thoughts (Bedewy & Gabriel, 2015; Casuso-Holgado et al., 2019; Olson et al., 2025). This heterogeneity of responses is particularly relevant in the university context, where academic demands may coincide with personal, social, and organizational changes characteristic of this stage (González Cabanach et al., 2018). 

Recent evidence suggests that, within the tripartite response system to stress, cognitive and emotional manifestations tend to be particularly pronounced among university students, especially in the form of persistent worry, intrusive negative thoughts, rumination, or emotional distress (Gil et al., 2023; Inostroza et al., 2024; Sheldon et al., 2021). Other studies have also emphasized the relevance of physical manifestations associated with academic demands, particularly when these demands are sustained over time or occur in highly demanding educational contexts (Bakker & Mostert, 2024; Frajerman et al., 2019; Madigan & Curran, 2021). However, physical responses to stress are not homogeneous: they may include both activation-related symptoms, such as physical agitation, and fatigue- or recovery-related symptoms, including exhaustion (Ghasemi et al., 2024). This variability should be understood as an expression of the idiosyncratic nature of academic stress: as Barraza Macías (2008) notes, each student expresses systemic imbalance through a specific pattern of indicators, both in type and intensity. On this basis, and rather than treating academic stress responses as a single global outcome, the present study focused on irritability, negative thoughts, and physical exhaustion as differentiated expressions of emotional–behavioral reactivity, cognitive interference, and recovery-related physical strain. 

Sleep disturbances have also come to occupy a prominent position in the study of university students’ well-being, affecting between 30% and 70% of this population (Becker et al., 2018; Cheng et al., 2012; Li et al., 2020). However, their relevance is not limited to their high prevalence but also extends to their relationship with emotional regulation, cognitive functioning, physical recovery, and academic performance (S. D. Hershner & Chervin, 2014; Lund et al., 2010). At the emotional–behavioral level, insufficient or poor-quality sleep has been linked to heightened emotional reactivity and weaker prefrontal regulation of amygdala-related responses, which may help account for irritability (Walker & van der Helm, 2009; Yoo et al., 2007). At the cognitive level, poor sleep may impair attentional control and executive functioning (Krause et al., 2017; Lima et al., 2022), while worry and rumination are consistently associated with poorer sleep, supporting a connection with negative thoughts (Clancy et al., 2020). At the physical level, disturbed sleep may compromise daytime functioning and restorative processes, contributing to fatigue and physical exhaustion (Shantakumari et al., 2024). Moreover, the university stage brings together conditions that are particularly conducive to the emergence of sleep disturbances, such as irregular schedules, late-night study habits, assessment pressure, and lifestyle changes characteristic of this period. In this regard, systematic reviews and meta-analyses have shown a consistent association between poor sleep quality, insomnia symptoms, and stress in university students (Gardani et al., 2022; Spyridonidis et al., 2025).

Beyond these response-specific links, available evidence suggests that sleep may be particularly compromised during periods of heightened academic pressure. Bouloukaki et al. (2023) found that examination periods were associated with poorer sleep quality and greater fatigue among university students. In addition, Kuhn et al. (2024) showed that sleep-related patterns may vary according to academic discipline, sleep hygiene, and dysfunctional beliefs about sleep. These findings place sleep disturbances in a relevant position within the academic stress process. Thus, sleep does not merely represent an isolated indicator of well-being but rather a psychophysiological dimension closely linked to how students cope with and experience academic demands (Dagani et al., 2024; Hu et al., 2024; Schmickler et al., 2023).

Against this background, the recent literature has begun to incorporate sleep quality into mediation models examining the association between academic stress and mental health. Among university students, academic stress has been found to be associated with indicators of distress both directly and through intermediate variables such as negative affect and sleep quality (Liu et al., 2023; X. Zhang et al., 2022). In line with this, recent studies in adolescent populations have examined academic stress–sleep–psychological health models in which sleep quality occupies an intermediate statistical position between academic stress and psychological health indicators (W. Zhang et al., 2026). Nevertheless, the available mediation evidence has mainly focused on general or internalizing indicators of mental health, such as depression or anxiety. Therefore, it remains necessary to clarify whether sleep disturbances are also involved in the association between academic stressors and specific academic stress responses, differentiated in their emotional–behavioral, cognitive, and physical expression.

**Figure 1:**
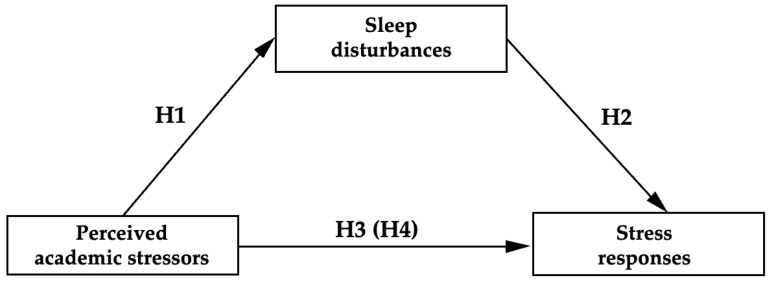
Conceptual model of the hypothesized associations.

Accordingly, the aim of the present study was to test a statistical mediation model in which sleep disturbances were specified as the statistical mediator between perceived academic stressors and three specific stress responses in university students: irritability, negative thoughts, and physical exhaustion. Based on the proposed model, the following hypotheses were formulated (Figure 1):

**H1.**  *Perceived academic stressors will be positively associated with sleep disturbances.*

**H2.**  *Sleep disturbances will be positively associated with irritability, negative thoughts, and physical exhaustion.*

**H3.**  *Perceived academic stressors will remain positively associated with irritability, negative thoughts, and physical exhaustion after accounting for sleep disturbances.*

**H4.**  *Sleep disturbances will partially account for the association between perceived academic stressors and irritability, negative thoughts, and physical exhaustion.*

## 2. Methods

### 2.1. Design and Participants

The study followed a quantitative, non-experimental, descriptive, and cross-sectional approach, framed within an ex post facto design (Ato et al., 2013). The reference population consisted of students enrolled in official undergraduate degree programs at the University of Extremadura during the 2024/2025 academic year and was estimated at approximately 18,000 students. A non-probability sampling procedure was used, with students’ academic groups serving as natural access units. The exact number of students approached within the selected academic groups was not systematically recorded; therefore, an exact participation rate could not be calculated. The minimum required sample size was estimated using this reference population, with a 95% confidence level, a 3% margin of error, and maximum variability (*p* = 0.50), resulting in a minimum of 1008 participants. The final valid sample comprised 1014 students, aged between 17 and 63 years (M = 20.56; SD = 3.50).

The inclusion criteria were being enrolled in an official undergraduate degree program at the University of Extremadura during the same academic year and voluntarily agreeing to participate in the study by providing informed consent. Students aged 17 were also eligible when participation was covered by the ethical conditions approved for the study.

Regarding gender distribution, 654 participants were women (64.5%) and 360 were men (35.5%). By year of study, 28.8% of the students were in their second year (*n* = 292), followed by first year (27.9%, *n* = 283), fourth year (23.3%, *n* = 236), and third year (20.0%, *n* = 203). Regarding field of study, the largest proportion of participants came from Social Sciences and Law (43.0%, *n* = 436), followed by Health Sciences (22.6%, *n* = 229), Sciences (17.2%, *n* = 174), Engineering and Architecture (8.8%, *n* = 89), and Arts and Humanities (8.5%, *n* = 86).

### 2.2. Measures

- Academic Stressors Scale (E-CEA; Cabanach et al., 2016). This instrument evaluates the frequency with which students perceive situations in the academic context as stressful. It consists of 54 items distributed across eight dimensions: methodological deficiencies of teachers, academic overload, beliefs about academic performance, public speaking, negative social climate, lack of value of contents, examinations, and participation difficulties. Responses are recorded on a 5-point Likert-type scale ranging from 1 (*never*) to 5 (*always*), with higher scores indicating a greater perception of academic stressors. Sample items include “*I feel nervous because of the excessive workload I have to deal with*,” “*I feel nervous when examination dates are approaching*,” and “*I feel nervous because I do not believe I can cope with the demands of the degree I am studying*.” A total score was computed, and internal consistency was high (Cronbach’s α = 0.91).

- Academic Stress Responses Scale (R-CEA; Cabanach et al., 2008). This instrument evaluates the frequency with which students experience symptoms associated with academic stress during recent weeks. It comprises 22 items rated on a 5-point Likert-type scale ranging from 1 (*never*) to 5 (*always*), with higher scores indicating a greater frequency of the corresponding symptoms. The scale includes five dimensions: sleep disturbances, negative thoughts, irritability, physical exhaustion, and physical agitation. For the purposes of the present study, the sleep disturbances dimension was specified as the mediating variable. Its six items address sleep initiation and maintenance difficulties, restless or non-restorative sleep, nocturnal or early awakenings, and disturbing dreams. A sample item is “*In recent weeks, I have difficulty falling asleep.*” Irritability, negative thoughts, and physical exhaustion were retained as outcome variables, each comprising four items. These dimensions assess, respectively, irritable or hostile reactions to setbacks, self-critical or intrusive negative thoughts, and fatigue-related symptoms. Representative items include “*In recent weeks, any setback irritates me*” for irritability, “*In recent weeks, I tend to emphasize my failures and devalue my successes*” for negative thoughts, and “*In recent weeks, I get tired easily*” for physical exhaustion. Internal consistency was adequate for the analyzed dimensions: sleep disturbances (α = 0.82), irritability (α = 0.87), negative thoughts (α = 0.85), and physical exhaustion (α = 0.80).

- Covariates. Sociodemographic and academic variables were collected using brief self-report items administered as part of the online survey. Gender was coded as 0 = men and 1 = women. Year of study was coded as 0 = early-year students (1st–2nd year) and 1 = later-years students (3rd–4th year). Field of study was coded as 0 = social sciences and humanities and 1 = science, technology, and health.

### 2.3. Procedure

Before data collection, faculties from different fields of knowledge at the University of Extremadura were contacted through institutional e-mails. The message provided information about the aims of the study, the content of the questionnaire, and the estimated completion time. Once institutional authorization had been obtained, the research team visited undergraduate classrooms in person and administered the survey collectively during regular class time, preserving the natural organization of students in their academic groups.

Data were collected between February and April of the 2024/2025 academic year and outside examination periods to reduce potential assessment-related bias. Students accessed the questionnaire through a QR code and completed it individually using Google Forms. The estimated completion time was approximately 20 min. Members of the research team remained present during administration to answer any procedural questions.

At the start of the questionnaire, students received information about the aims of the study, the voluntary nature of participation, the exclusive use of the data for scientific purposes, and their right to decline participation or withdraw before submitting the questionnaire without any consequences. Anonymity and confidentiality were guaranteed, and no personal identifying information was requested. Participants were also informed that there were no right or wrong answers to reduce social desirability bias. Digital informed consent was required before accessing the questionnaire.

The online form was configured with mandatory response fields for all items; therefore, incomplete responses could not be submitted. After the survey period, the database was reviewed to identify possible inconsistencies or out-of-range values. Cases containing evident response errors or inconsistencies were excluded during data screening, representing less than 1% of the submitted responses.

The study was conducted in accordance with the principles of the Declaration of Helsinki and the Ethical Code of the University of Extremadura.

### 2.4. Data Analysis

For data analysis, descriptive statistics were first computed for the main study variables. Internal consistency was examined using Cronbach’s alpha, and Pearson bivariate correlations were estimated as preliminary analyses. Subsequently, three statistical mediation models were estimated using PROCESS Model 4 (Hayes, 2022) in IBM SPSS Statistics, version 26.0. Within the mediation framework, academic stressors were specified as the independent variable and sleep disturbances as the statistical mediator. Three separate models were estimated, one for each stress response outcome: irritability, negative thoughts, and physical exhaustion. Gender, year of study, and field of study were included as covariates in all models. Standardized regression coefficients are reported, together with standard errors, t values, 95% confidence intervals, R^2^, and omnibus F statistics for the regression equations underlying the mediation models.

The significance of indirect effects was examined using bootstrapped 95% confidence intervals based on 5000 resamples. Indirect effects were considered statistically significant when the confidence interval did not include zero. Standardized direct, indirect, and total effects were reported for each model. For descriptive purposes, the proportion of the total association represented by each pathway was also reported. The level of statistical significance was set at *p* < 0.05.

## 3. Results

### 3.1. Initial Analyses

Table 1 summarizes the descriptive statistics and bivariate correlations for the primary study variables. All variables showed positive and statistically significant associations. Academic stressors were moderately to strongly associated with sleep disturbances and with the three stress responses. Sleep disturbances were also positively related to irritability, negative thoughts, and physical exhaustion, and the stress responses were significantly intercorrelated.

### 3.2. Mediation Models

Three statistical mediation models were estimated to examine whether sleep disturbances accounted for part of the associations between academic stressors and three stress responses: irritability, negative thoughts, and physical exhaustion.

As shown in Table 2, the model for sleep disturbances was statistically significant, F = 93.20, *p* < 0.001, and explained 27% of the variance in sleep disturbances. Academic stressors were positively associated with sleep disturbances (β = 0.52, t = 20.00, *p* < 0.001). The outcome models were also statistically significant: irritability, F = 118.40, *p* < 0.001; negative thoughts, F = 186.10, *p* < 0.001; and physical exhaustion, F = 201.60, *p* < 0.001. These models explained 37%, 48%, and 50% of the variance, respectively. Within these outcome models, sleep disturbances were positively associated with irritability (β = 0.36, t = 12.00, *p* < 0.001), negative thoughts (β = 0.30, t = 10.00, *p* < 0.001), and physical exhaustion (β = 0.41, t = 13.67, *p* < 0.001). Academic stressors also remained positively associated with irritability (β = 0.32, t = 10.32, *p* < 0.001), negative thoughts (β = 0.48, t = 15.48, *p* < 0.001), and physical exhaustion (β = 0.40, t = 12.90, *p* < 0.001).

Among the covariates, gender was positively associated with sleep disturbances (β = 0.10, *p* = 0.017) and with the three stress responses (β = 0.07–0.14, all *p* < 0.05), indicating higher scores among women. Field of study was negatively associated with sleep disturbances (β = −0.06, *p* = 0.039) and physical exhaustion (β = −0.05, *p* = 0.046), indicating lower scores among students in science, technology, and health fields compared with those in social sciences and humanities. Year of study was negatively associated only with negative thoughts (β = −0.06, *p* = 0.033), indicating lower scores among later-year students compared with early-year students. No other covariate associations reached statistical significance.

Standardized direct, indirect, and total effects are summarized in Table 3. All estimates were adjusted for gender, year of study, and field of study. After accounting for sleep disturbances, academic stressors remained positively associated with irritability, negative thoughts, and physical exhaustion. Evidence of indirect associations through sleep disturbances was observed in the three models, with 95% bootstrap confidence intervals excluding zero. This indirect pathway accounted for 37.25% of the total association with irritability, 25.00% with negative thoughts, and 34.43% with physical exhaustion. Overall, the findings are consistent with a pattern of partial cross-sectional statistical mediation across the three adjusted models.

**Figure 2:**
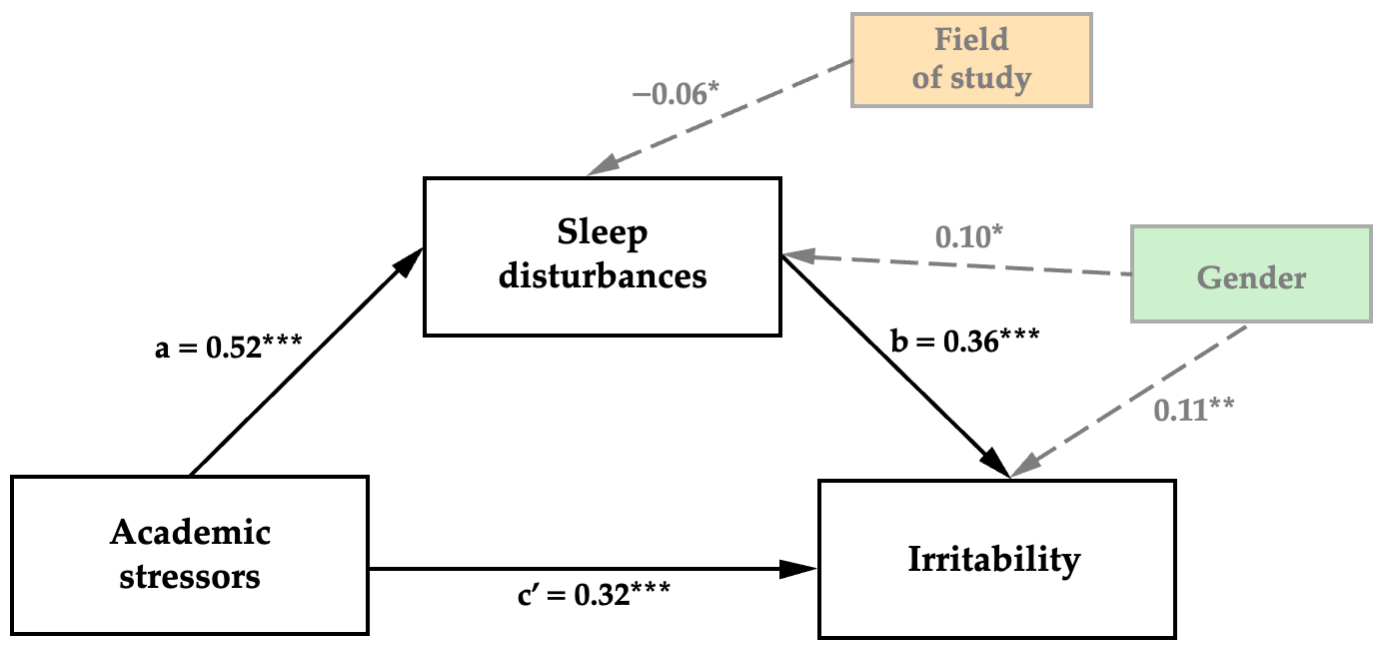
Representation of Mediation Model 1. Note. Solid arrows represent paths among the main study variables, whereas dashed arrows represent covariate paths. Only statistically significant covariate effects are shown. * *p* < 0.05; ** *p* < 0.01; *** *p* < 0.001.

**Figure 3:**
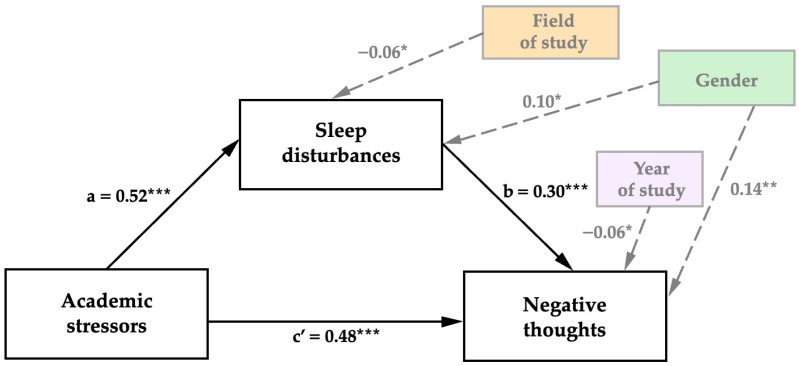
Representation of Mediation Model 2. Note. Solid arrows represent paths among the main study variables, whereas dashed arrows represent covariate paths. Only statistically significant covariate effects are shown. * *p* < 0.05; ** *p* < 0.01; *** *p* < 0.001.

**Figure 4:**
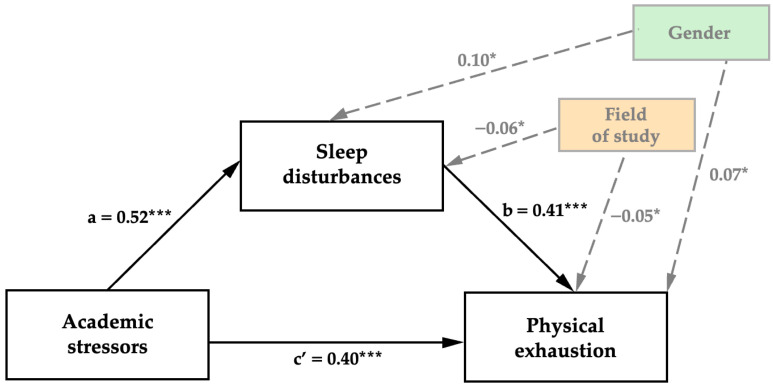
Representation of Mediation Model 3. Note. Solid arrows represent paths among the main study variables, whereas dashed arrows represent covariate paths. Only statistically significant covariate effects are shown. * *p* < 0.05; *** *p* < 0.001.

Figure 2, Figure 3 and Figure 4 provide a visual representation of the three adjusted mediation models, showing the associations among academic stressors, sleep disturbances, and each stress response. To improve readability, only statistically significant covariate paths are displayed.

## 4. Discussion

The present study tested a statistical mediation model in which sleep disturbances were specified as the statistical mediator between perceived academic stressors and three specific stress responses in university students: irritability, negative thoughts, and physical exhaustion. Overall, the findings showed a consistent pattern across the three adjusted models. Academic stressors were positively associated with sleep disturbances, and sleep disturbances were, in turn, associated with higher levels of irritability, negative thoughts, and physical exhaustion. In addition, the indirect effects through sleep disturbances were statistically significant in all models, while the direct effects of academic stressors on the three outcomes remained significant. This pattern is consistent with partial cross-sectional statistical mediation, indicating that sleep disturbances accounted for part—but not all—of the association between perceived academic stressors and differentiated stress responses. The outcome models explained 37%, 48%, and 50% of the variance in irritability, negative thoughts, and physical exhaustion, respectively, suggesting a meaningful proportion of explained variance, particularly for the cognitive and physical response models.

In relation to the first hypothesis, higher levels of academic stressors were associated with greater sleep disturbances. This finding is consistent with recent evidence indicating that university students’ sleep is sensitive to study-related strain and lifestyle conditions, including academic workload, perceived stress, exhaustion, and irregular sleep routines (AlHamlan et al., 2025; Schmickler et al., 2023). It also converges with systematic evidence linking perceived stress with poorer sleep quality and insomnia symptoms in undergraduate students (Gardani et al., 2022; Nakie et al., 2024). In addition, studies focused on periods of heightened academic pressure, such as examinations, have shown poorer sleep quality, greater fatigue, and poorer perceived academic functioning (Bouloukaki et al., 2023; Suardiaz-Muro et al., 2023). In this context, sleep disturbances may be interpreted as a stress-related response associated with academic demands that interfere with recovery processes and regular sleep routines.

The second hypothesis was also supported: sleep disturbances were positively associated with the three stress responses analyzed. This result is theoretically consistent, given that sleep contributes to emotional regulation, cognitive functioning, and physical recovery. The association between sleep disturbances and irritability may reflect the role of insufficient or disrupted sleep in reducing emotion-regulation capacity and increasing reactivity to situations perceived as frustrating or demanding (Ben Simon et al., 2020). Previous evidence has linked poor sleep to emotional dysregulation, greater negative affect, and increased vulnerability to irritability or emotional instability (Palmer & Alfano, 2017; Walker & van der Helm, 2009). In the educational context, this association may be particularly relevant, as academic demands require sustained emotional adjustment in response to tasks, assessments, and performance-related pressures (Tomaso et al., 2021). Thus, sleep disturbances may lower the threshold for frustration and favor more reactive responses, which is consistent with the positive association with irritability observed in the model.

Sleep disturbances were also associated with negative thoughts. This finding is consistent with evidence linking poor sleep to repetitive negative thinking, rumination, and cognitive-emotional vulnerability (Clancy et al., 2020; Inostroza et al., 2024). Sleep disruption may impair cognitive control, increase attentional bias toward threatening or negative information, and reduce students’ ability to disengage from academic concerns (Cox et al., 2018; Nota & Coles, 2018). In this sense, when cognitive activation associated with academic demands extends beyond the immediate academic situation—particularly during periods intended for rest or phases of heightened academic pressure—difficulties falling asleep or maintaining sleep may coexist with intrusive academic worries, self-critical thoughts, and anticipatory concerns about performance (Amaral et al., 2018; Benham, 2021). Together, these findings suggest a reciprocal cognitive pattern: academic worries may persist into periods intended for rest, while persistent worry, rumination, and intrusive academic concerns may also interfere with sleep (Petak & Maričić, 2025).

The association between sleep disturbances and physical exhaustion was also significant and, descriptively, the strongest of the three sleep–response associations. This pattern is consistent with the restorative function of sleep, as disrupted rest may be associated with reduced physical recovery, greater daytime fatigue, lack of energy, and perceived exhaustion. In university populations, previous studies have shown that poor sleep quality is related to higher levels of fatigue, particularly in contexts of high academic demand (Bouloukaki et al., 2023; Inan et al., 2025), while broader models of student well-being have emphasized that insufficient rest and recovery may contribute to symptoms related to academic strain or burnout under sustained academic demands (Bakker & Mostert, 2024; Madigan & Curran, 2021). The findings of the present study therefore suggest that sleep disturbances may be especially relevant for understanding the physical component of academic stress, particularly physical exhaustion. Given the conceptual proximity between sleep, fatigue, and recovery, this association should be interpreted cautiously and not as evidence that either process temporally precedes the other.

With regard to the third hypothesis, perceived academic stressors maintained significant direct effects on irritability, negative thoughts, and physical exhaustion after accounting for sleep disturbances; therefore, the third hypothesis was supported. This finding indicates that sleep disturbances did not fully account for the association between perceived academic stressors and the three stress responses analyzed. This pattern is consistent with previous evidence showing that academic stress is associated with poorer mental well-being, greater distress, and burnout-related symptoms in university students (Barbayannis et al., 2022; Olson et al., 2025). The persistence of these direct effects may reflect the multifaceted nature of academic stressors and their connection with processes beyond sleep disruption, such as perceived overload, evaluative pressure, appraisal of control, emotional strain, or insufficient academic resources (Bakker & Mostert, 2024; Bedewy & Gabriel, 2015).

The fourth hypothesis was also supported in statistical terms, as indirect associations through sleep disturbances were observed across the three models. Specifically, the indirect component represented 37.25% of the total association with irritability, 25.00% with negative thoughts, and 34.43% with physical exhaustion. This pattern is consistent with recent mediation studies in which sleep quality has been specified as an intermediate variable in models relating academic stress to psychological health indicators (Liu et al., 2023; X. Zhang et al., 2022). Taken together, these findings suggest that sleep disturbances represented a meaningful, although partial, component of the association between perceived academic stressors and differentiated stress responses.

The inclusion of covariates provided additional nuances to the main pattern of findings. Gender was positively associated with sleep disturbances and the three stress responses, suggesting slightly higher levels among women; this pattern aligns with previous evidence on gender differences in sleep problems, fatigue, and psychological distress among students (B. Zhang & Wing, 2006; Zeng et al., 2020), although the effects were small. Field of study was negatively associated with sleep disturbances and physical exhaustion, indicating slightly lower levels among students in science, technology, and health than in social sciences and humanities. However, this result should be interpreted cautiously, as field-related differences may depend on sample composition, academic routines, assessment formats, and discipline-specific demands (Kuhn et al., 2024; Olson et al., 2025). Finally, year of study showed a small negative association with negative thoughts, which may be interpreted in light of the adjustment demands and stress-related vulnerability often characterizing the initial stages of university life (March-Amengual et al., 2022). Overall, these covariate effects were small and did not alter the main pattern of findings linking academic stressors, sleep disturbances, and differentiated stress responses. 

### 4.1. Theoretical and Practical Implications

From a theoretical perspective, the present study contributes to the literature on academic stress by examining sleep disturbances as a specific dimension within the academic stress-response system that shows a distinctive statistical role relative to other response dimensions. Because sleep disturbances belong to the same response framework as irritability, negative thoughts, and physical exhaustion, the findings should not be interpreted as treating sleep as external to stress responses, but rather as situating it within a differentiated response system. This contribution reinforces a process-oriented and dimensional understanding of academic stress, highlighting the value of examining specific emotional–behavioral, cognitive, and physical manifestations rather than reducing academic stress responses to broad, undifferentiated indicators (Barraza Macías, 2008).

At the applied level, these findings may be particularly relevant for university contexts similar to the one examined here, underscoring the value of incorporating sleep-related components into stress-prevention and health-promotion programs. At the individual level, psychoeducational initiatives focused on sleep hygiene, the regularization of study and rest routines, and awareness of the association between sleep and academic stress may be useful, particularly during periods of high academic demand. In this regard, previous evidence suggests that sleep education and sleep-focused interventions can improve sleep habits, sleep quality, and other indicators of well-being in university students (S. Hershner & O’Brien, 2018; Saruhanjan et al., 2021). However, these results should not be interpreted as placing responsibility exclusively on students. Given that perceived academic stressors maintained direct effects on the three stress responses, institutional actions should also address structural and organizational aspects of the academic environment, such as coordinated assessment planning, a balanced distribution of workload, early detection of excessive academic pressure, and policies that support healthier study and rest patterns. Thus, sleep health should be integrated into broader institutional strategies for academic well-being, rather than framed as an isolated recommendation operating solely at the individual level.

### 4.2. Limitations and Future Research

Despite these contributions, several limitations should be acknowledged. First, the cross-sectional design precludes causal or temporal conclusions; therefore, future longitudinal studies should examine the sequence linking academic stressors, sleep disturbances, and stress responses, as well as potential reciprocal relationships between sleep disturbances and academic stress. Second, the data were collected through self-report measures, which are appropriate for assessing perceived academic stress and symptoms but may be susceptible to response biases and common method variance, particularly because sleep disturbances and the three stress-response outcomes were assessed at the same time point using subscales of the same academic stress-response instrument. Third, sleep disturbances were assessed using an embedded subscale rather than a sleep-specific instrument, which does not allow for a clinical or multidimensional assessment of sleep quality or insomnia symptoms. Future research could incorporate sleep-specific instruments and, where possible, objective or ecological measures of sleep.

In addition, academic stressors were analyzed as a global score, which may obscure the differential contribution of specific demands, such as academic overload, examinations, or beliefs about academic performance. Similarly, year of study and field of study were included as broad covariates, so more fine-grained differences across individual years of study or disciplinary areas could not be examined. Future studies should consider retaining these variables in more detailed categories when sample size and model complexity allow. A further methodological consideration is that students were recruited through naturally occurring academic groups; therefore, some degree of clustering at the class-group level cannot be ruled out. Future studies could use multilevel designs to account for the nested structure of students within academic groups.

Finally, the use of non-probability sampling and the single-university sample, drawn from a public university in southwestern Spain, may limit the generalizability of the findings to other institutional, cultural, or educational contexts. Local academic organization, assessment schedules, daily routines, and rest patterns may shape how students experience academic demands, sleep disturbances, and stress responses. Therefore, the findings should be interpreted primarily in relation to similar university contexts. Replication across different universities and academic systems would be advisable, as would examining whether variables such as age, gender, year of study, or field of study moderate the mediation process. It would also be valuable to test longitudinal or multilevel models during specific periods of the academic calendar, such as examination weeks, to gain a more precise understanding of how academic demands, sleep disturbances, and stress responses interact over time.

## 5. Conclusions

The present study highlights the relevance of sleep disturbances within the academic stress-response system. The findings are consistent with partial statistical mediation, showing that perceived academic stressors were associated with irritability, negative thoughts, and physical exhaustion both directly and indirectly through sleep disturbances. By focusing on differentiated emotional–behavioral, cognitive, and physical responses, the study reinforces the need to move beyond broad indicators of student stress and to consider how specific response dimensions are interrelated within the university context.

Taken together, these results point to the need for prevention strategies operating at both individual and institutional levels. Sleep-focused actions, such as sleep hygiene education and the promotion of regular study–rest routines, may be useful, particularly during periods of high academic demand. However, sleep health should not be framed solely as an individual responsibility. In university contexts similar to the one examined here, institutional policies should also address structural sources of academic strain, including workload concentration, assessment pressure, limited coordination of academic demands, and insufficient opportunities for recovery. Integrating sleep health into broader academic well-being strategies may therefore contribute to more sustainable learning environments and better support students’ adjustment and well-being throughout university life.

## Figures and Tables

**Table 1:** Descriptive statistics and correlation matrix.

Variables	*M* (*SD*)	1.	2.	3.	4.	5.
1. Academic stressors	2.70 (0.67)	1				
2. Sleep disturbances	2.37 (0.81)	0.51 **	1			
3. Irritability	2.41 (0.89)	0.53 **	0.54 **	1		
4. Negative thoughts	2.72 (1.02)	0.63 **	0.54 **	0.55 **	1	
5. Physical exhaustion	2.94 (0.95)	0.61 **	0.61 **	0.55 **	0.58 **	1

Note. *M* = mean; *SD* = standard deviation. Scores ranged from 1 to 5. ** *p* < 0.01.

**Table 2:** Regression coefficients for the mediation models.

Criterion Variable	Predictor	β	SE	t	95% CI LL	95% CI UL	R^2^	F
Sleep disturbances	Academic stressors	0.52	0.026	20.00	0.47	0.57	0.27	93.20
	Gender	0.10	0.042	2.38	0.02	0.18		
	Year of study	−0.02	0.030	−0.67	−0.08	0.04		
	Field of study	−0.06	0.029	−2.07	−0.12	−0.003		
Irritability	Academic stressors	0.32	0.031	10.32	0.26	0.38	0.37	118.40
	Sleep disturbances	0.36	0.030	12.00	0.30	0.42		
	Gender	0.11	0.038	2.89	0.04	0.18		
	Year of study	−0.03	0.030	−1.00	−0.09	0.03		
	Field of study	−0.02	0.029	−0.69	−0.08	0.04		
Negative thoughts	Academic stressors	0.48	0.031	15.48	0.42	0.54	0.48	186.10
	Sleep disturbances	0.30	0.030	10.00	0.24	0.36		
	Gender	0.14	0.046	3.04	0.05	0.23		
	Year of study	−0.06	0.028	−2.14	−0.11	−0.01		
	Field of study	−0.04	0.028	−1.43	−0.09	0.02		
Physical exhaustion	Academic stressors	0.40	0.031	12.90	0.34	0.46	0.50	201.60
	Sleep disturbances	0.41	0.030	13.67	0.35	0.47		
	Gender	0.07	0.030	2.33	0.01	0.13		
	Year of study	−0.03	0.029	−1.03	−0.09	0.03		
	Field of study	−0.05	0.025	−2.00	−0.10	−0.001		

Note. β = standardized coefficient; SE = standard error; t = *t* statistic; CI = confidence interval; LL = lower limit; UL = upper limit. R^2^ = coefficient of determination; F = omnibus *F* statistic. Effects whose 95% CI did not include zero were considered statistically significant.

**Table 3:** Standardized direct, indirect, and total effects of the mediation models.

Effect	Path	β	95% CI LL	95% CI UL	Proportion
Direct effects (c′)					
Model 1	Academic stressors → Irritability	0.32	0.26	0.38	62.75%
Model 2	Academic stressors → Negative thoughts	0.48	0.42	0.54	75.00%
Model 3	Academic stressors → Physical exhaustion	0.40	0.34	0.46	65.57%
Indirect effects (ab)					
Model 1	Academic stressors → Sleep disturbances → Irritability	0.19	0.15	0.23	37.25%
Model 2	Academic stressors → Sleep disturbances → Negative thoughts	0.16	0.12	0.20	25.00%
Model 3	Academic stressors → Sleep disturbances → Physical exhaustion	0.21	0.17	0.25	34.43%
Total effects (c)					
Model 1	Academic stressors → Irritability	0.51	0.45	0.57	
Model 2	Academic stressors → Negative thoughts	0.64	0.58	0.70	
Model 3	Academic stressors → Physical exhaustion	0.61	0.55	0.67	

Note. β = standardized coefficient; CI = confidence interval. c′ = direct effect after accounting for the indirect pathway through sleep disturbances; ab = indirect effect through sleep disturbances; c = total effect before decomposing the association into direct and indirect effects. For indirect effects, CIs are bootstrapped. Proportion (%) refers to the percentage of the total effect represented by each direct or indirect effect and is therefore reported only for direct and indirect effects.

## Data Availability

The data are available upon request from the corresponding author.
